# Empowering local communities using artificial intelligence

**DOI:** 10.1016/j.patter.2022.100449

**Published:** 2022-03-11

**Authors:** Yen-Chia Hsu, Ting-Hao ‘Kenneth’ Huang, Himanshu Verma, Andrea Mauri, Illah Nourbakhsh, Alessandro Bozzon

**Affiliations:** 1 Faculty of Industrial Design Engineering, Delft University of Technology, Delft, the Netherlands; 2 College of Information Sciences and Technology, Pennsylvania State University, State College, PA, USA; 3 Robotics Institute, Carnegie Mellon University, Pittsburgh, PA, USA; 4 Amsterdam Institute for Advanced Metropolitan Solutions, Amsterdam, the Netherlands

**Keywords:** artificial intelligence, community citizen science, community empowerment, human-computer interaction, social impact, sustainability, applied research

## Abstract

Artificial intelligence (AI) applications can profoundly affect society. Recently, there has been extensive interest in studying how scientists design AI systems for general tasks. However, it remains an open question as to whether the AI systems developed in this way can work as expected in different regional contexts while simultaneously empowering local people. How can scientists co-create AI systems with local communities to address regional concerns? This article contributes new perspectives in this underexplored direction at the intersection of data science, AI, citizen science, and human-computer interaction. Through case studies, we discuss challenges in co-designing AI systems with local people, collecting and explaining community data using AI, and adapting AI systems to long-term social change. We also consolidate insights into bridging AI research and citizen needs, including evaluating the social impact of AI, curating community datasets for AI development, and building AI pipelines to explain data patterns to laypeople.

## Highlights

• Co-creating AI systems can empower local communities to address regional concerns
• Designing AI for social impact is the key to linking AI research closer to local needs
• Curating data with local people can yield agency to them and facilitate AI research
• Explaining data patterns using AI can reveal local issues for public scrutiny

## The bigger picture

Artificial intelligence (AI) is increasingly used to analyze large amounts of data in various practices, such as object recognition. We are specifically interested in using AI-powered systems to engage local communities in developing plans or solutions for pressing societal and environmental concerns. Such local contexts often involve multiple stakeholders with different and even contradictory agendas, resulting in mismatched expectations of the behaviors and desired outcomes of these systems. There is a need to investigate whether AI models and pipelines can work as expected in different contexts through co-creation and field deployment. Based on case studies in co-creating AI-powered systems with local people, we explain challenges that require more attention and provide viable paths to bridge AI research with citizen needs. We advocate for developing new collaboration approaches and mindsets that are needed to co-create AI-powered systems in multi-stakeholder contexts to address local concerns.

## Teaser

AI systems have great potential to be applied in achieving Sustainable Development Goals. However, conflicts of interest among multiple stakeholders result in challenges when co-designing AI systems with local people, collecting community data to fine-tune the AI models, and adapting the behavior of AI to long-term social change. Through case studies and the literature, this article explains these challenges and highlights viable paths toward empowering local communities to advocate for social and policy changes in response to pressing regional issues.

## Introduction

Artificial intelligence (AI) techniques are typically engineered with the goals of high performance and accuracy. Recently, AI algorithms have been integrated into diverse and real-world applications. Exploring the impact of AI on society from a people-centered perspective has become an important topic.^1^ Previous works in citizen science have identified methods of using AI to engage the public in research, such as sustaining participation, verifying data quality, classifying and labeling objects, predicting user interests, and explaining data patterns.^2^, ^3^, ^4^, ^5^ These works investigated the challenges regarding how scientists design AI systems for citizens to participate in research projects at a large geographic scale in a generalizable way, such as building applications for citizens globally to participate in completing tasks. In contrast, we are interested in another area that receives significantly less attention: *How can scientists co-create AI systems with local communities to address context-specific concerns and influence a particular geographic region?*

Our perspective is based on applying AI in Community Citizen Science^6^^,^^7^ (CCS), a framework to create social impact through community empowerment at an intensely place-based local scale. We define *community* as a group of people who are indirectly or directly affected by issues in civil society and are dedicated to making sure that these issues are recognized and resolved. We define *social impact* as how a project influences the society and local communities that are affected by social or environmental issues. We define *community empowerment* as a process of yielding agency to communities so that they can use technology, data, and informed rhetoric to create and disseminate evidence to advocate for social and policy changes. The CCS framework, a branch of citizen science,^8^^,^^9^ is beneficial in co-creating solutions and driving social impact with communities that pursue the Sustainable Development Goals.^10^ Based on the literature and our experiences in co-creating AI systems with citizens, this article provides critical reflections regarding this underexplored topic for data science, AI, citizen science, and human-computer interaction fields. We discuss the challenges and insights in connecting AI research closely to social issues and citizen needs, using prior works as examples.

### How CCS links to other frameworks

CCS emphasizes close collaborations among stakeholders when tackling local concerns. It is inspired by community-based participatory research^11^ and popular epidemiology,^12^ in which citizens directly engage in gathering data and extract knowledge from these data for advocacy and activism. Examples involve co-designing technology for local watershed management,^13^ understanding water quality with local communities,^14^^,^^15^ and using geo-information tools to monitor noise and earthquakes.^16^ CCS intends to extend the scope of previous frameworks to Sustainable Development Goals, especially the goal of sustainable cities and communities. This article discusses using CCS to integrate AI in-the-wild and local regions, which is different from those that conducted studies in living lab environments (e.g., the work by Alavi et al.^17^) or in online communities (e.g., the work by Brambilla et al.^18^).

In addition, CCS is related to action research,^19^ RtD (research through design),^20^ service design,^21^ and the PACT (participatory approach to enable capabilities in communities) framework.^22^ Extending action research, CCS encourages scientists to immerse themselves in the field by taking on a social role and conducting research from a first-person view. Complementing RtD that creates prototypes as proof-of-concept, CCS develops functional systems that can be deployed and used by local people. Unlike service design, citizens’ roles extend beyond service consumers to co-designers who co-create knowledge and systems with scientists and other stakeholders. The PACT framework and CCS share the same goal of co-designing AI systems to address critical societal issues, while CCS has an additional goal that needs to be achieved simultaneously: empowering local communities to catalyze social impact.

### Challenges

Due to its region-based characteristics, CCS often involves many local stakeholders—including communities, citizens, scientists, designers, and policy makers—with complex relationships. CCS creates the space for the stakeholders to reveal underlying difficulties and locally oriented action plans in tackling social concerns that are hard to uncover in traditional technology-oriented and researcher-centered approaches. However, stakeholders often have divergent and even contradicting values, which result in conflicts that pose challenges when designing, engineering, deploying, and evaluating AI systems.

**Figure 1:**
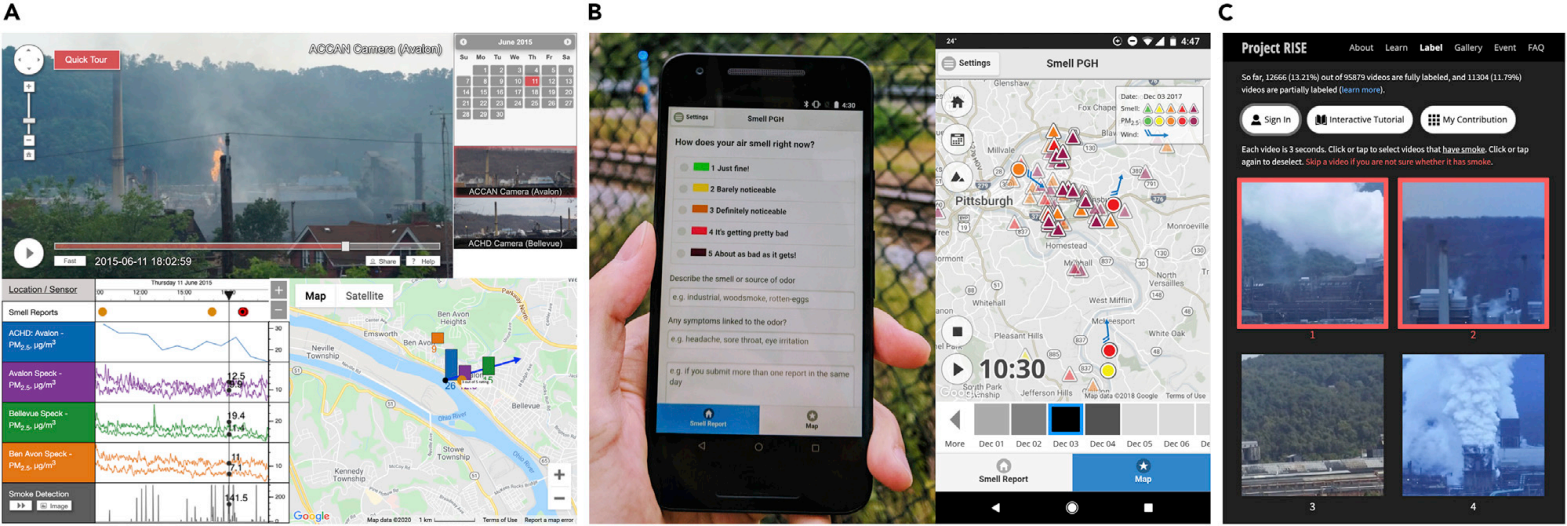
Case studies of community citizen science projects that involve co-designing AI tools with local communities (A) The air pollution monitoring project^23^ that empowered the Pittsburgh community to collect air pollution evidence in the local region for taking action. (B) The Smell Pittsburgh Project^24^ invited citizens to report pollution odors and use the data as evidence to conduct air pollution studies. (C) The RISE project^25^ enabled citizens and scientists to annotate industrial smoke emissions and build an AI model to recognize pollution events. These cases were approved by the ethics committee of the university that hosted the projects.

Based on case studies (Figure 1) of collaborating with local people in building AI systems, we outline three major challenges:

• Co-designing AI systems with local communities
• Collecting and explaining community data using AI
• Adapting AI systems to long-term social changes

These challenges come mainly from the conflicts of interest between local communities (e.g., citizens, community groups) and university researchers (e.g., designers, scientists). AI researchers pursue knowledge to advance science, while local communities often desire social change. Such conflicts of interest among these two groups can lead to tensions, socio-technical gaps, and mismatched expectations when co-designing and engineering AI systems. For instance, local communities need functional and reliable systems to collect evidence, but AI researchers may be interested in producing system prototypes only to prove concepts or answer their research questions. Local community concerns can be urgent and timely, and citizens need to take practical actions that can have an immediate and effective social impact, such as public policy change. However, scientists need to produce knowledge using rigorous methods and publish papers in the academic community, often requiring a long reviewing and publication cycle.

### Co-designing AI systems with local communities

Challenges exist in community co-design, especially when translating multifaceted community needs to implementable AI system features without using research-centered methods. The current practice to design AI systems is mainly centered on researchers instead of local people. Popular research methods, such as participatory design workshops, interviews, and surveys, are normally used to help designers and scientists understand research questions. Although these methods enable researchers to better control the research process, essentially, university researchers are privileged and in charge of the conversations, leading to inappropriate power dynamics that can hinder trust and exacerbate inequality.^26^^,^^27^ For example, during our informal conversations with local people that suffer from environmental concerns in our air quality monitoring project,^23^ many expressed feelings that scientists often treated them as experimental subjects (but not collaborators) in research studies. This imbalanced power relationship leads to difficulties in initiating conversations with citizens during our community outreach efforts.

Also, community data and knowledge are hyperlocal, which indicates that their underlying meanings ground closely to the local region and could be difficult to grasp for researchers who are not a part of the local community.^28^ For example, citizen-organized meetings to discuss community actions are often dynamic and context-dependent, which is not designed nor structured for research purposes. To collect research data that represent community knowledge, the current intensive procedure, such as video or audio recording, can make citizens feel uncomfortable. One alternative is to be a part of the community, to design solutions with them, join their actions, and perform ethnographic observations. For instance, researchers can better understand local community needs by actively participating in regional citizen group meetings and daily conversations with citizens. However, such in-depth community outreach approaches take tremendous personal effort, which can be unmotivating or even infeasible due to the limited academic research cycle and research-oriented academic tenure awarding system.^11^^,^^27^

### Collecting and explaining community data using AI

Challenges arise in data collection, analysis, and interpretation due to conflicts of interest among scientists and citizens. Scientists look for rigorous procedures, but citizens seek evidence for action. Local communities are often frustrated by the formal scientific research procedure to prove the adverse impact of risk,^12^ such as finding evidence of how pollution negatively affects health. Traditional environmental risk assessment models require a causal link between the risk and the outcome with statistical significance before taking action, which can be very difficult to achieve due to complex relationships between local people and their environments.^29^ As a result, citizens collect their own community data (as defined by Carroll et al.,^30^ such as photographs of smoke emission from a nearby factory) as an alternative to prove their hypotheses. From scientists’ point of view, however, such strong assumption-driven evidentiary collection can lead to biases since the collection, annotation, and analysis of community data are conducted in a manner that strongly favors the assumption. One example is confirmation bias, in which people are incentivized to search for information and provide data that confirm their prior beliefs,^31^ such as a high tendency to report odors related to pollution events.^24^ Based on our experiences, it is extremely difficult to address or eliminate such biases when analyzing and interpreting how local social or environmental concerns affect communities.

Furthermore, researchers need to evaluate the social impact of AI systems to understand whether the community co-design approach is practical. However, it is hard to determine whether the intervention of AI systems actually influences the local people and leads to social changes by statistically analyzing community data. One difficulty is that people may have the implicit cognitive bias to overestimate and overstate the effect of the intervention since they are deeply involved in the co-creation of the AI system.^32^ Moreover, it can be infeasible to conduct randomized experiments to prove the effectiveness of the intervention of AI on local communities.^7^ Controlling volunteer demographics and participation levels can be unethical when analyzing impact among different groups of people. CCS treats local people as collaborators rather than as participants. Researchers in CCS take the supporting role to assist communities using technology, instead of supervising and overseeing the entire project.^7^ Therefore, citizens join the CCS project at will and are not recruited as in typical research studies. AI systems, in this case, are deployed in the wild with real consequences rather than a controlled test-bed environment that is designed for hypothesis testing. It remains an open research question as to how to integrate social science when studying the impact of AI systems.^33^

### Adapting AI systems to long-term social changes

Conflicts of interest in the diverged values of citizens and scientists can lead to challenges in adapting AI systems to long-term social changes. The relationship between local people and AI systems is a feedback loop, which is similar to the concept that human interactions with architectural infrastructure are a continuous adaptation process that spans over long periods of time.^34^ When embedded in the social context, AI systems interact with citizens daily as community infrastructure. Communities are dynamic and frequently evolve their agenda to adapt to social context changes. This means that the AI systems also need to adjust to such changes in local communities continuously. For instance, as we understand more about the real-life effects of the deployed AI systems on local people, we may need to fine-tune the underlying machine learning model using local community data. We may also need to improve the data analysis pipeline and strategies for interpreting results to fit local community needs in taking action. We may even need to stop the AI system from intervening in the local community under certain conditions. Such adaptation at scale requires ongoing commitment from researchers, designers, and developers to continuously maintain the infrastructure, involve local people in assessing the impact of AI, adjust the behavior of AI systems, and support communities in taking action to advocate for social changes.^35^

However, it is very challenging to estimate and obtain the required resources to sustain such long-term university-community engagement with local people,^36^ especially in financially supporting local community members for their efforts. Typical research procedures can be laborious in data collection and analysis, and engineering AI systems with local people requires a tremendous community outreach effort to establish mutual trust. In our experiences, applying and evaluating AI in CCS relies heavily on an environment that has a sustainable fundraising mechanism in community organizations and universities. For example, funding is needed to hire software engineers that can maintain AI systems as community infrastructure in the long term, which can be hard to achieve in the current academic grant instruments and funding cycles.

The success of CCS also depends on sustainable participation, which requires high levels of altruism, high awareness of local issues, and sufficient self-efficacy among local people. However, the complexity of the underlying machine learning techniques can affect the willingness to participate. On one side is whether the automation technique is trustworthy. In our experience, local communities often perceive AI as a mysterious box that can be questionable and is not always guaranteed to work. Hence, the willingness of citizens to provide data can be low, but AI systems that use machine learning and computer vision need data to be functional. On the other side, “ what citizens think the AI system can do” does not match “what the AI system can actually do,” resulting in socio-technical gaps and pitfalls for actual usage.^37^ In our experience, local communities often have high expectations about what AI techniques can do for them—for example, automatically determining whether an industrial site is violating environmental regulations. However, in practice, the AI system may only identify whether a factory emits smoke and degrades the quality of the air through sensors and cameras, which requires additional human efforts to verify whether the pollution event is indeed a violation.

### Bridge AI research and citizen needs

University researchers typically lead the development of AI systems using a researcher-centered approach, in which they often have more power over local communities (especially underserved ones) in terms of scientific authority and available resources. This unequal power relationship can result in a lack of trust and cause harm to underserved communities.^26^^,^^36^ An underlying assumption of this researcher-centered approach is that designers and scientists can put themselves in the situation of citizens and empathize with local people’s perspectives. However, university researchers are in a privileged situation in terms of socio-economic status and may come from other geographical regions or cultures, which means it can be very challenging for researchers to fully and authentically understand local people’s experiences.^12^ Only by admitting this weakness and recognizing the power inequality can researchers truly respect community knowledge and be sincerely open-minded in involving local communities—especially those affected by the problems the most—in the center of the design process when creating AI systems. Beyond being like the local people and designing solutions for them, researchers need to be with people who are affected by local concerns to co-create historicity and ensure that the AI systems are created to be valuable and beneficial to them.^35^^,^^38^

The critical role of creating social impact lies in local people and their long-term perseverance in advocating for changes. We believe that scientists need to collaborate with local people to address pressing social concerns genuinely, and even further, to immerse themselves into the local context and become citizens, hence "scientific citizens" (as defined by Irwin^8^). However, pursuing academic research and addressing citizen concerns require different (even contradictory) efforts and can be difficult to achieve at the same time. Academic research requires contributing papers with scientific knowledge primarily to the research community, while citizen concerns typically involve many other stakeholders in a large and regional socio-technical system. It remains an open question how scientists and citizens can collaborate effectively under such dynamic, hyperlocal, and place-based conditions.^39^

To move forward, we propose three viable CCS approaches about how AI designers and scientists can conduct research and co-create social impact with local communities:

• Evaluate social impact of AI as empirical contributions
• Curate community data as dataset contributions
• Build AI pipelines as methodological contributions

These approaches produce empirical, dataset, and methodological contributions, respectively, to the research community, as defined by Wobbrock and Kientz.^40^ To the local people, these approaches establish a long-term fair university-community partnership in addressing community concerns, increase literacy in collecting community data, and equip communities with AI tools to interpret data. CCS projects will succeed when designers and scientists see themselves as citizens, and in turn, when local communities and citizens see themselves as innovators. It is essential for all parties to collaborate around the lived experiences of one another and listen to each other’s voices with humility and respect.

### Social impact of AI as empirical contributions

Lessons learned from previously deployed AI systems in other contexts cannot be simply applied in the current one, as local communities have various cultures, behaviors, beliefs, values, and characteristics.^33^ Hence, it is essential to understand and document how scientists can co-design AI systems with local communities and co-create long-term social impact in diverse contexts. It is also important to study the effectiveness and impact of various AI interventions with different design criteria in sustaining participation, affecting community attitude, and empowering people. The CCS framework provides a promising path toward these goals. Implications of collaborating with local people in co-designing AI interventions, creating long-term effects, and tackling the conflicts of interest among stakeholders can be strong empirical contributions to the academic community.^35^ The data-driven evidence and the interventions that are produced by AI systems can affect the local region in various ways, including increasing residents’ confidence in addressing concerns, providing convincing evidence, or rebalancing power relationships among stakeholders.

For instance, our air pollution monitoring project documented the co-design process in regard to how designers translated citizen needs and local knowledge into implementable AI system features, as recognized by the human-computer interaction community and published in the proceedings of the Association for Computing Machinery Conference on Human Factors in Computing Systems (ACM CHI).^23^ This work shows researchers how we co-created an AI system to support citizens in collecting air pollution evidence and how local communities used the evidence to take action. For example, in the computer vision model for finding industrial smoke emissions in videos, the feature vectors are handcrafted according to the behaviors and characteristics of smoke, which are provided by community knowledge. Also, the communities decide the areas in the video that require image processing. The decision of having high precision in the prediction (instead of high recall) is also a design choice by local people for quickly determining severe environmental violations. Another example is our study of push notifications, which are generated by an AI model to predict the presence of bad odors in the city.^24^ The finding from the study explains how sending certain types of push notifications to local citizens is related to the increase of their engagement level, such as contributing more odor reports or browsing more data.

Although one may not simply duplicate the collaboration ecosystem in these contexts due to unique characteristics in the local communities, our projects can be seen as case studies in specific settings. Our air quality monitoring case provides insights to researchers working on similar problems in other contexts about integrating technology reliably into their settings, as cited by Ottinger.^41^ Moreover, the case also helps researchers understand and categorize different modes of community empowerment, as cited by Schneider et al.^42^

### Community data as dataset contributions

Data work is critical in building and maintaining AI systems,^43^ as modern AI models are powered by large and constantly changing datasets. When addressing local concerns with the support of AI systems, researchers often need to fine-tune existing models or build new pipelines to fit local needs. This requires collecting data in a specific regional context and may introduce new tasks to the AI research field. CCS provides a sustainable way to co-create high-quality regional datasets while simultaneously increasing citizens’ self-efficacy in addressing local problems. Based on our experiences, co-creating publicly available community data can also facilitate citizens’ sense of ownership of the collaborative work. This value of community empowerment links AI research closely to social impact and the public good.

Besides the value of increasing citizens’ data literacy, the collected real-world data, the data collection approach, and the data processing pipeline can be combined into a significant dataset contribution to the academic community in creating robust AI models. Such community datasets are gathered in the wild with local populations over a long period to reflect the regional context, which complements the datasets obtained using crowdsourcing approaches (e.g., Amazon Mechanical Turk) in a broader context. In this way, community datasets provide values for AI researchers to validate whether AI models trained on general datasets can work as expected in different regional contexts. Also, the accompanying software for data labeling can contribute reusable computational tools to the research community that investigates data annotation strategies.

For example, our RISE (Recognizing Industrial Smoke Emissions) project presented a novel video dataset for the smoke recognition task, which can help other researchers develop better computer vision models for similar tasks, as recognized by the AI community and published by the Association for the Advancement of Artificial Intelligence (AAAI).^25^ Our project demonstrated the approach of collaborating with citizens affected by air pollution to annotate videos with industrial smoke emissions on a large scale under various weather and lighting conditions. The dataset was used to train a computer vision model to recognize industrial smoke emissions, which allowed community activists to curate a list of severe pollution events as evidence to conduct studies and advocate for enforcement action. Another example is the Mosquito Alert project, which curates and labels a large mosquito image dataset with local people using a mobile application.^44^ The dataset is built with local community knowledge and is used to train a mosquito recognition model to support the local public health agency in disease management. In addition to its social impact, the Mosquito Alert project advances science by providing a real-world dataset for researching different mosquito recognition models, as cited by Adhane et al.^45^

### AI pipelines as methodological contributions

In CCS, there is a need to unite expertise from the local communities and scientists to build AI pipelines using machine learning to assist data labeling, predict trends, or interpret patterns in the data. An example is to forecast pollution and find evidence of how pollution affects the quality of living in a local region. Although the concept of machine learning is common among computer scientists, it can seem mysterious to citizens.

Thus, during public communication and community outreach, researchers often need to visualize analysis results and explain the statistical evidence for local residents, which is highly related to the explainable AI (XAI) and interpretable machine learning research.^46^ However, current XAI research focuses mainly on making AI understandable for experts rather than laypeople and local communities.^47^ This creates a unique research opportunity to study co-design methods and software engineering workflows of translating the predictions of AI models and their internal decision-making process into human-intelligible insights in the hyperlocal context.^1^^,^^48^^,^^49^ We believe the pipeline of such translation into explainable evidence can be a methodological contribution to the academic community, which provides a way to address the challenge of predicting future trends and interpreting similar types of real-world data. Also, the implemented machine learning pipeline and the design insights of developing the pipeline can contribute reusable computational tools and novel software engineering workflows to the research communities that study XAI and its user interfaces.

For instance, our Smell Pittsburgh project used machine learning to explain relationships between citizen-contributed odor reports and air quality sensor measurements, contributing to a methodological pipeline of translating AI predictions, as recognized by the intelligent user interface community and published in the ACM Transactions on Interactive Intelligent Systems (TiiS).^24^ In this way, the pollution patterns became visible to public scrutiny and debate. Another example is the xAire project, which co-designed solutions with local schools and communities to collect nitrogen dioxide data.^50^ Air measurements were analyzed with asthma cases in children using a statistical machine learning model. The community outreach and public communication enabled laypeople to make sense of how nitrogen dioxide posed a risk to local community health. The pipelines in these two examples produced meaningful patterns for citizens to understand and communicate about how pollution affects the local region. They also informed researchers about how to process, wrangle, analyze, and interpret urban data to explain insights to laypeople.

### Next steps

We have explained major challenges in co-designing AI systems with local people and empowering them to create a broader social impact. We also proposed CCS approaches to simultaneously addressing local societal issues and advancing science. Computing research communities have taken steps to recognize the impact of technology and AI on society, such as establishing a separate track for paper evaluation (e.g., the AAAI Special Track on AI for Social Impact). We urge the computing research communities to go further and acknowledge social impact as a type of formal contribution in scientific inquiry and paper publication. Promoting this kind of contribution can be a turning point to encourage scientists to link research to society and ultimately make university research socially responsible for the public good. Co-creating AI systems and developing reusable tools with local communities in the long term allows scientists and designers to explore real-world challenges and solution spaces for various AI techniques, including machine learning, computer vision, and natural language processing.

We also urge universities to integrate social impact into the evaluation criteria of the tenure roadmap of the academic professorship as the “service” pillar of the university that contributes to the public good. We envision that applying CCS when co-designing AI systems can advance science, build public trust in AI research through genuine reciprocal university-community partnership, and directly support community action to affect society. In this way, we may fundamentally change how universities, organizations, and companies partner with their neighbors to pursue shared prosperity in the future of community-empowered AI.
